# Effect of Physical Activity Participation on Lifestyle Habits and School Life Among Korean Children

**DOI:** 10.3390/children12050570

**Published:** 2025-04-28

**Authors:** Seungok An, Su-Yeon Roh, Jeonga Kwon

**Affiliations:** 1 Department of Special Physical Education, Yongin University, Yongin 17092, Republic of Korea; skyforve@yiu.ac.kr; 2 Department of Exercise Rehabilitation, Gachon University, Incheon 21936, Republic of Korea; 3 Department of Elementary Education, College of First, Korea National University of Education, Cheongju 28173, Republic of Korea

**Keywords:** adolescents, lifestyle habits, physical activity, school life

## Abstract

*Objectives:* This study explored the impact of physical activity (PA) participation on the lifestyle habits and school life of Korean elementary school students. *Methods:* We collected survey data from 28,514 elementary school students participating in the 2023 Student Health Examination conducted by the Korea Ministry of Education. PA participation was the independent variable, defined as whether elementary school students participate in exercise that makes them out of breath or sweat more than three times a week. The variables related to lifestyle habits included breakfast intake, amount of sleep, TV viewing, gaming/Internet use, thoughts about running away from home, perceived body image, and body mass index. The variables for school life included experiences of being bullied, the need for counseling regarding school life problems, and the need for counseling for distress. The collected data were analyzed using frequency analysis, chi-squared tests, and multivariate logistic regression analyses. *Results:* PA was significantly associated with regular breakfast intake, sufficient sleep, limited television viewing, reduced gaming/Internet use, and a positive perception of body image. Specifically, regarding breakfast intake, the average odds ratio (OR) was 1.160 for always having it. Regarding the amount of sleep, the average OR was 0.836 for less than 6 h, 0.692 for 6–7 h, and 0.767 for 7–8 h. Regarding TV viewing, the average OR was 0.831 for yes. For gaming, the average OR was 0.770 for yes. Regarding perceived body image, the average OR was 1.429 for slightly thin, 1.487 for normal, and 1.400 for slightly fat. *Conclusions:* These results suggest that children’s PA facilitates the formation of good lifestyle habits; therefore, it should be actively encouraged in children.

## 1. Introduction

Childhood is an important period of life. During this time, an individual acquires the physical, cognitive, emotional, social, and economic resources that lay the foundation for health and well-being in later years [1]. This phase is particularly salient for disease prevention and health promotion in adulthood [2]. Furthermore, studies have shown that habits such as poor nutrition, reduced exercise, obesity, alcohol consumption, tobacco use, poor mental health, and interpersonal violence can begin as early as childhood and adolescence [3]. Therefore, it is imperative to address these issues during childhood to prevent their continuation into adulthood. As the Korean proverb “a habit formed at the age of three lasts until the age of eighty” suggests, habits formed in childhood can turn into lifelong habits. This is particularly relevant to physical activity (PA), as regular PA in childhood can lead to lifelong engagement. PA refers to the movement of skeletal muscles that requires energy expenditure [4]. The American College of Sports Medicine’s minimum exercise guidelines recommend at least 20 min of aerobic activity and one set of 8–10 resistance exercises (8–12 repetitions) on three days per week, targeting major muscle groups on two of those days [5]. In this study, PA was defined as exercising to the point of breathlessness or sweating more than three times a week. This definition of PA is both appropriate and comprehensive. Bélanger et al. [6] assessed the relationship between PA levels during childhood and adulthood among Canadian children aged 12–13. They found that PA in childhood leads to higher PA levels in adulthood [6]. Malina et al. [7] tracked people’s PA and physical fitness from childhood to adulthood and observed that PA and physical fitness habits in childhood continue into adulthood and affect health [7]. Thus, it can be concluded that PA habits formed during childhood continue into adulthood and contribute to a healthy life.

To live a healthy life, it is important to develop a healthy lifestyle. Regular PA, proper eating habits, good posture, positive thinking, appropriate media use, and sufficient sleep contribute to a healthy lifestyle [8,9]. However, how children’s participation in PA affects their lifestyle remains unclear. Previous studies have found that PA facilitates the formation of proper eating habits [10,11], improves sleep quality [12,13], reduces media use [14,15], fosters emotional development [16,17], and aids in obesity prevention [18,19]. Thus, it can be inferred that children’s PA fosters a healthy lifestyle and positively affects adult life.

Another vital question is how children’s engagement in PA affects their school life. Studies have reported that children’s PA positively impacts their social skills, peer relationships, and learning attitudes [20,21,22]. Liu et al. [20] conducted a meta-analysis on the relationship between children’s PA and bullying. They found a significant negative correlation between PA and bullying victimization [20]. The positive association between bullying and PA arises from the opportunity for children to engage in PA with friends, allowing them to learn essential virtues and values for coexisting with others, such as cooperation, care, respect, and forgiveness.

Kwon et al. [21] analyzed the relationship between adolescents’ PA, learning attitudes, and interpersonal skills. They reported that the more adolescents engaged in PA, the better their learning attitudes and interpersonal skills were [21]. Chen et al. [22] reported that children’s participation in PA enhanced their attention and concentration. Thus, it can be concluded that children’s engagement in PA significantly affects their peer relationships and academic performance, contributing to a smoother school experience.

By and large, it can be inferred that PA helps children have a healthy lifestyle and smooth school life, thereby positively affecting their growth and development. However, previous studies on the relationship between children’s PA and lifestyle habits and school life have analyzed these relationships separately. It is necessary to analyze how children’s PA is related to their lifestyle habits (such as eating habits, sleep habits, and media usage) and school life (e.g., friendships and worries). The development of healthy lifestyle habits and active participation in school life during childhood are crucial, as they notably influence children’s lives. Research related to PA among elementary school students has mainly focused on its effects on their health and learning. Healthy lifestyle habits and school life are important because they affect the well-being of elementary school students. This study differs from previous research by examining, in detail, the mid- to long-term impact of PA participation among Korean elementary school students on their health and lifestyle habits. Therefore, this study aimed to explore the effects of PA on lifestyle habits and school life among Korean elementary school students. The results of this study can be used as foundational data for creating a healthy lifestyle and desirable school life among children. Specifically, these data can help develop health policies for children and for elementary school teachers to guide children. The results of this study will be valuable for research on children’s growth and development. The research questions of this study are as follows: First, does participation in PAs positively impact Korean children’s lifestyle habits? Second, does participation in PAs positively affect Korean children’s school life?

## 2. Materials and Methods

### 2.1. Design, Study Population, and Data Source

This study used survey data from the 2023 Student Health Examination conducted by the Korea Ministry of Education. This survey focuses on PA, school life problems, class attitudes, bullying, hyperactivity, and attention span among elementary, middle, and high school students. A total of 87,183 students participated in the 2023 Student Health Examination. After excluding the data of middle and high school students and those who did not respond, we utilized the data of 28,514 elementary school students. The Korea Ministry of Education selects sample schools and sends questionnaires to them for students to respond. The sample schools send the survey responses to the Korea Ministry of Education through the National Education Information System. The Korea Ministry of Education collects the responses and publishes them on its website. We entered our personal information, such as sex, age, occupation, and email address, into the Korea Ministry of Education website and used the secondary data for research. The 2023 Student Health Examination was approved by the Institutional Review Board of the Korea Ministry of Education (Number: 112002) and conducted according to the principles outlined in the Declaration of Helsinki. The survey results were posted (in Korean) on the website for academic purposes after deleting personally identifiable information “https://schoolhealth.kr/web/srs/selectPrivacyAgree.do?popup=true#none (accessed on 7 February 2025)”. All participants and their guardians were informed of the study’s purpose, and they voluntarily signed an informed consent form.

### 2.2. Measures

#### 2.2.1. Independent Variable

The independent variable was participation in PA. It was measured using the question “Do you exercise to the point of breathlessness or sweating more than three times a week”? The response options were yes (1 point) and no (2 points). We used these responses without any modifications.

#### 2.2.2. Dependent Variables

The dependent variables were lifestyle habits and school life factors. The lifestyle habits variables were breakfast intake, amount of sleep, TV viewing, gaming/Internet use, thoughts about running away from home, perceived body image, and body mass index (BMI). The school life variables were the experience of being bullied, the need for counseling for school life problems, and the need for counseling for distress.

Breakfast intake was measured by asking respondents “Do you eat breakfast”? The response options were always (1 point), usually (2 points), usually not (3 points), and never (4 points). The amount of sleep was assessed using the question “How many hours do you usually sleep per day”? The response options were less than 6 h (1 point), 6–7 h (2 points), 7–8 h (3 points), and more than 8 h (4 points). TV viewing was measured by asking respondents, “Do you watch television for more than 2 h a day”? The response options were yes (1 point) and no (2 points). Gaming/Internet use was assessed using the question “Do you spend more than 2 h a day on the Internet or playing games”? The response options were yes (1 point) and no (2 points). Thoughts about running away from home were measured by asking respondents “Do you often feel like running away from home”? The response options were yes (1 point) and no (2 points). Perceived body image was measured using the question “How do you think your body shape is compared to those of your friends”? The response options were very thin (1 point), slightly thin (2 points), normal (3 points), slightly fat (4 points), and very fat (5 points). BMI was measured using the item “Please enter your height and weight” and was calculated using the following formula: BMI = (weight)/(height)^2^. Then, participants were categorized as underweight (<18.5 kg/m^2^), normal (18.5–<23 kg/m^2^), overweight (23–<25 kg/m^2^), or obese (≥25 kg/m^2^).

The experience of being bullied was assessed using the question “Have you been bullied or ostracized by your friends in the past year”? The response options were yes (1 point) and no (2 points). The need for counseling for school life problems was assessed by asking respondents “Do you need counseling from a teacher because of the problems you face at home or school”? The response options were yes (1 point) and no (2 points). The need for counseling for distress was measured using the question “Do you need counseling because of worries or distress”? The response options were yes (1 point) and no (2 points). We used all responses, except for BMI, without any modifications.

#### 2.2.3. Covariate

The covariate was sex, categorized as male or female. In this study, 14,665 (51.4%) were males and 13,849 (48.6%) were females.

### 2.3. Data Analysis

The collected data were analyzed as follows: First, we conducted a frequency analysis of all participant characteristics. Frequency analysis is the most basic statistical technique for examining the frequency of variables. This study was conducted to objectively determine the distribution of variations. Second, we performed chi-squared tests to identify differences in participant characteristics based on PA participation. Chi-squared tests are statistical techniques used to assess the association between two variables. This was conducted to examine the relationship between the variables used in this study. Third, we conducted multivariate logistic regression analyses to examine the associations between PA, breakfast intake, amount of sleep, TV viewing, gaming/Internet use, thoughts of running away from home, perceived body image, BMI, experience of being bullied, the need for counseling for school-related problems, and the need for counseling for distress. Multivariate logistic regression is a statistical technique used to determine the relationship between independent and dependent variables. The study was conducted to determine the effect of PA participation on lifestyle habits and school life among Korean children. Odds ratios (ORs), 95% confidence intervals (CIs), and *p*-values were calculated. All statistical analyses were performed using SPSS for Windows (version 23.0; IBM Corp., Armonk, NY, USA), and statistical significance was set at *p* < 0.05.

## 3. Results

### 3.1. Participant Characteristics

Table 1 presents data on participant characteristics. Of the 28,514 respondents, there were 14,665 (51.4%) males and 13,849 (48.6%) females. Respondents engaged in PA (65%). Regarding lifestyle habits, most respondents reported that they always ate breakfast (57.0%) and slept for more than 8 h per day (54.6%). The highest number of respondents perceived their body shape as normal (42.8%), while most respondents were identified as underweight (56.2%). Furthermore, most respondents answered “no” to questions regarding TV viewing, gaming/Internet use, and thoughts about running away from home. Concerning school life, most respondents indicated that they had not experienced bullying and did not require counseling for school life problems or distress.

### 3.2. Differences in Participant Characteristics Based on PA

Table 2 presents the results of the chi-squared tests. Sex (χ2 = 819.991, *p* < 0.001), breakfast intake (χ2 = 114.717, *p* < 0.001), amount of sleep (χ2 = 214.761, *p* < 0.001), TV viewing (χ2 = 84.918, *p* < 0.001), gaming/Internet use (χ2 = 182.497, *p* < 0.001), thoughts about running away from home (χ2 = 0.261, *p* = 0.618), perceived body image (χ2 = 59.003, *p* < 0.001), BMI (χ2 = 1.376, *p* = 0.711), experience of being bullied (χ2 = 5.559, *p* = 0.019), the need for counseling for school life problems (χ2 = 0.407, *p* = 0.533), and the need for counseling for distress (χ2 = 6.244, *p* = 0.014) differed significantly based on participation in PA. In summary, sex, breakfast intake, amount of sleep, TV viewing, gaming/Internet use, perceived body image, experiences of bullying, and the need for counseling for distress were statistically significant.

### 3.3. Association Between Participation in PA and Lifestyle Habits

Table 3 presents the results of the multivariate logistic regression analysis examining the association between participation in PA and lifestyle habits. Regarding breakfast intake, the average OR was 1.160 (95% CI: 1.057–1.272; *p* = 0.002) for always, 1.062 (95% CI: 0.963–1.171; *p* = 0.231) for usually, and 0.955 (0.853–1.070; *p* = 0.426) for usually not. Regarding the amount of sleep, the average OR was 0.836 (95% CI: 0.709–0.986; *p* = 0.034) for less than 6 h, 0.692 (95% CI: 0.632–0.758; *p* < 0.001) for 6–7 h, and 0.767 (95% CI: 0.726–0.810; *p* < 0.001) for 7–8 h. Regarding TV viewing, the average OR was 0.831 (95% CI: 0.786–0.878; *p* < 0.001) for yes. Regarding gaming/Internet use, the average OR was 0.770 (95% CI: 0.729–0.813; *p* < 0.001) for yes. Regarding thoughts about running away from home, the average OR was 1.099 (95% CI: 0.958–1.260; *p* = 0.178) for yes. For perceived body image, the average OR was 1.138 (95% CI: 0.978–1.323; *p* = 0.094) for very thin, 1.429 (95% CI: 1.247–1.637; *p* < 0.001) for slightly thin, 1.487 (95% CI: 1.312–1.686; *p* < 0.001) for normal, and 1.400 (95% CI: 1.242–1.579; *p* < 0.001) for slightly fat. As for BMI, the average OR was 0.945 (95% CI: 0.842–1.061; *p* = 0.341) for underweight, 0.980 (95% CI: 0.878–1.093; *p* = 0.712) for normal, and 0.962 (95% CI: 0.842–1.100; *p* = 0.574) for overweight. In summary, it was confirmed that participation in PA positively influenced breakfast intake, amount of sleep, TV viewing, gaming/Internet use, and perceived body image.

### 3.4. Association Between Participation in PA and School Life Factors

Table 4 presents the results of the multivariate logistic regression analysis examining the association between participation in PA and school life factors. Regarding the experience of being bullied, the average OR was 0.878 (95% CI: 0.764–1.008; *p* = 0.065) for yes. Regarding the need for counseling for school life problems, the average OR was 1.125 (95% CI: 0.934–1.355; *p* = 0.215) for yes. Regarding the need for counseling for distress, the average OR was 0.802 (95% CI: 0.635–1.013; *p* = 0.064) for yes. In summary, participation in PA was not statistically significant in relation to the school life factor.

## 4. Discussion

This study yielded several insightful results. First, participation in PA was significantly related to regular breakfast consumption among elementary school students. Elementary school students who engaged in PA were more likely to eat breakfast every day than to never eat breakfast. This finding aligns with the results of previous studies [10,11,23]. Vissers et al. [10] explored the relationship between breakfast intake and PA among British children aged 9–10 years. They found that children exhibited a stronger relationship between breakfast intake and PA, which was consistent with the findings of this study.

Zakrzewski-Fruer et al. [11] explored the relationship between breakfast frequency and PA among children in 12 countries. They reported that the more frequently breakfast was consumed, the greater the participation was in moderate-to-vigorous PA [11]. Yoshimura et al. [23] examined the relationship between breakfast consumption and PA among adults. Their results partially align with those of the present study, indicating that skipping breakfast leads to decreased PA and increased energy intake among adult women, resulting in a higher BMI [23]. It is crucial for children to eat breakfast regularly. Regular breakfast consumption enhances children’s cognitive abilities, supports balanced growth, and helps prevent obesity [24]. Most studies included in the systematic review have shown that children and adolescents who regularly skip breakfast are more likely to be overweight and have a higher overall energy intake than those who regularly eat breakfast [25,26]. Given that regular breakfast consumption helps prevent obesity, it is important to establish the habit of regular breakfast consumption from an early age [25,26]. Our findings substantiate that participation in PA influences the development of healthy breakfast habits and is beneficial for children’s health.

Second, PA was significantly related to sufficient sleep among elementary school students. Elementary school students who engaged in PA were less likely to sleep 6–7 or 7–8 h per day than 8 h per day. This result suggests that participation in PA contributes to sufficient sleep among elementary school students. Moreover, this finding is consistent with previous studies [12,13]. Larrinaga-Undabarrena et al. [12] analyzed the relationship between levels of PA and sleep among children and adolescents aged 6–17 in the Basque Country. They found that higher levels of PA were associated with better sleep efficiency. Ekstedt et al. [13] analyzed the relationship between PA and sleep quality among Swedish children aged 6–10 and reported that intensive PA during the day improved sleep quality. However, some studies have found no correlation or a negative correlation between PA and sleep [27,28]. Eythorsdottir et al. [27] investigated the relationship between PA and sleep habits among 54 children aged 2–6 years. They observed that high levels of PA were associated with poor sleep habits. Vincent et al. [28] studied the relationship between PA and sleep among children aged 8–11 years and found no temporal or bidirectional correlation between the two variables. It is important for children to get sufficient sleep, as it has a positive effect on their cognitive, physical, and psychological health, thus benefiting their growth and development [29]. International guidelines recommend 9–11 h of sleep for children. Roman-Viñas et al. [30] investigated sleep duration among children from 12 countries and found that out of 7372 children, only 42% met this recommendation. Sleep problems in children can be caused by numerous factors, and PA is a notable way to address these issues [31]. Our results support the notion that PA can be an effective method for alleviating sleep problems in children.

Third, PA was significantly related to not watching TV and no gaming/Internet use among elementary school students. Elementary school students who participated in PA were more likely not to watch TV and not to engage in gaming/Internet use for more than 2 h per day. This result is consistent with that of previous studies [14,15,32]. Dutra et al. [14] conducted a cohort study on the relationship between PA and TV viewing among 616 8-year-old children. They observed that TV viewing duration per day was inversely related to PA levels. Mineshita et al. [15] investigated the relationship between Internet usage duration and PA among 7419 Japanese elementary school students. They found that prolonged Internet usage could lead to decreased PA. Chan et al. [32] analyzed the relationship between PA and e-sports and online video games among adolescents. They reported that participation in e-sports and video games not only reduced PA among adolescents but also hampered the formation of good lifestyle habits [32].

Many studies have shown that increased time spent on apps, social media, games, and the Internet using televisions, computers, smartphones, and tablets is related to poorer cardio-metabolic health, shorter sleep duration, increased risk of obesity, and negative effects on mental health in children [33]. In particular, the correlation between TV viewing and obesity in children may be mediated by a lack of PA [34]. Screen use has increased considerably worldwide, often at the expense of PA. In fact, many recent studies have reported that children’s PA has decreased while their TV viewing and gaming/Internet use have increased [35]. During the COVID-19 pandemic, increased sedentary behavior led to decreased physical fitness in children [36]. Sedentary behaviors, such as watching TV and playing games, decrease physical fitness, regardless of the level of PA [37]. Today, PA can serve as a mediator for children, helping them build physical fitness and reduce sedentary behavior [35,38]. This is because PA can directly reduce sedentary behavior and energy intake. Our results confirm that participation in PA may help reduce sedentary behavior among children.

Fourth, PA was significantly related to perceiving one’s body image as thin or normal among elementary school students. Hausenblas et al. [39] conducted a meta-analysis of the effects of PA on perceived body image. They found that participants who exercised had more positive perceptions of their body image than those who did not, and their body image scores significantly improved after the exercise intervention [39]. This suggests that engaging in PA positively affects perceived body image. It is important to have positive perceptions of one’s body image, as body image satisfaction is linked to a positive self-concept, psychological well-being, and healthy social relationships, all of which contribute to a good quality of life [40,41]. During childhood and adolescence, individuals undergo significant physical changes and experience mental processes that heighten the awareness of their bodies and increase their tendency to compare themselves with others [42]. This period is crucial for developing a healthy sense of body image, and older adolescents tend to have more negative perceptions of their body image than younger adolescents [42]. In this context, our results demonstrate that PA is an effective means of enhancing children’s positive perceptions of their body image, as well as their emotional well-being.

Fifth, the relationship between PA and school life was not statistically significant. García-Hermoso et al. [43] meta-analyzed the relationship between PA and bullying victimization in children and adolescents. Their results differed from this study in that they reported that bullying victimization rates were higher when PA guidelines were not followed [43]. In addition, Carter et al. [44] conducted a systematic review and meta-analysis of the effects of PA on distress in children and adolescents. Their results also differed from this study, as they reported that PA was potentially effective in reducing distress in children and adolescents [44]. Moreover, Opstoel et al. [45] reported that PA in children and adolescents had a positive effect on personal and social development. PA in children and adolescents improves factors necessary for them to have harmonious relationships with others and a peaceful school life, such as prosocial behavior, cooperation, communication, and problem-solving skills [45]. In other words, the results of this study, which showed that the relationship between children’s PA and school life was not statistically significant, differed from those of previous studies.

This study has some limitations. First, as this was a secondary study, it has limitations in explaining temporal causality. Additionally, this research aimed to ensure the success of a large-scale study that would closely examine the research question. No validity or reliability testing of the survey instrument used was conducted. The results of this study should be interpreted cautiously, given the limitations in explaining the causal relationship between PA and each variable of lifestyle habits and school life. Second, this study did not use actual measurements of lifestyle habits or PA. There may have been recall or response bias in the responses related to lifestyle habits and school life. For example, the number of times one consumed breakfast, the number of hours of sleep per day, regular participation in PA, nor height and weight were measured objectively. If the variables had been measured using objective methods, the results of this study may have differed. Third, the PA measure used in this study was only a single question. In addition, the PA examples included walking, cycling, and swimming, and the intensity of PA was not confirmed. Therefore, the question about PA was not accurate. In addition, only one question was used for each variable, including lifestyle habits, school life, and PA; thus, the responses may not have represented the true expression of each variable. Therefore, if multiple questions had been used for each variable, the results may have been different. Fourth, in this study, lifestyle habits included not only breakfast intake and amount of sleep but also perceived body image and BMI, which are a result of lifestyle habits. Thus, in this study, lifestyle habits included not only the behaviors of elementary school students but also the consequences of these habits. Fifth, the study population consisted of elementary school students in Korea; therefore, the results cannot be generalized. Furthermore, the results of this study may differ depending on the demographic characteristics of the study population. Therefore, there is a limitation in generalizing them to children worldwide. Future studies should expand the scope of the study population.

## 5. Conclusions

PA was significantly related to regular breakfast intake, sufficient sleep, limited television viewing, minimal gaming/Internet use for prolonged periods, and having positive perceptions of one’s body image. Good lifestyle habits must be established to lead a healthy life. In particular, it is important that such habits are formed as early as childhood, as habits developed during this time tend to persist throughout life. Children’s participation in PA promotes the development of good lifestyle habits; therefore, their PA should be actively encouraged.

## Figures and Tables

**Table 1:** Participant characteristics (n = 28,514).

Characteristic	Categories	n (%)
Sex	Male	14,665 (51.4%)
	Female	13,849 (48.6%)
Participation in physical activity	Yes	18,526 (65.0%)
	No	9988 (35.0%)
Breakfast intake	Always	16,255 (57.0%)
	Usually	7015 (24.6%)
	Usually not	2848 (10.0%)
	Never	2396 (8.4%)
Amount of sleep	Less than 6 h	671 (2.4%)
	6–7 h	2490 (8.7%)
	7–8 h	9787 (34.3%)
	More than 8 h	15,566 (54.6%)
TV viewing	Yes	8536 (29.9%)
	No	19,978 (70.1%)
Gaming/Internet use	Yes	10,714 (37.6%)
	No	17,800 (62.4%)
Thoughts about running away from home	Yes	1023 (3.6%)
	No	27,491 (96.4%)
Perceived body image	Very thin	2383 (8.3%)
	Slightly thin	6032 (21.2%)
	Normal	12,219 (42.8%)
	Slightly fat	6316 (22.2%)
	Very fat	1564 (5.5%)
Body mass index (kg/m^2^)	Underweight	16,031 (56.2%)
	Normal	8443 (29.6%)
	Overweight	1887 (6.6%)
	Obese	2153 (7.6%)
Experience of being bullied	Yes	964 (3.4%)
	No	27,550 (96.6%)
Need for counseling for school life problems	Yes	562 (2.0%)
	No	27,952 (98.0%)
Need for counseling for distress	Yes	348 (1.2%)
	No	28,166 (98.8%)

**Table 2:** Differences in participant characteristics based on participation in physical activity (n = 28,514).

Characteristic	Categories	Physical Activity	No physical Activity	χ^2^ (*p*)
Sex	Male	10,681 (57.7%)	3984 (39.9%)	819.991 (<0.001 ***)
	Female	7845 (42.3%)	6004 (60.1%)	
Breakfast intake	Always	10,950 (59.1%)	5305 (53.1%)	114.717 (<0.001 ***)
	Usually	4451 (24.0%)	2564 (25.7%)	
	Usually not	1695 (9.2%)	1153 (11.5%)	
	Never	1430 (7.7%)	965 (9.7%)	
Amount of sleep	Less than 6 h	427 (2.3%)	244 (2.4%)	214.761 (<0.001 ***)
	6–7 h	1428 (7.7%)	1062 (10.6%)	
	7–8 h	5992 (32.4%)	3795 (38.1%)	
	More than 8 h	10,679 (57.6%)	4887 (48.9%)	
TV viewing	Yes	5206 (28.1%)	3330 (33.3%)	84.918 (<0.001 ***)
	No	13,320 (71.9%)	6658 (66.7%)	
Gaming/Internet use	Yes	6434 (34.7%)	4280 (42.9%)	182.497 (<0.001 ***)
	No	12,092 (65.3%)	5708 (57.1%)	
Thoughts about running away from home	Yes	657 (3.5%)	366 (3.7%)	0.261 (0.618)
	No	17,869 (96.5%)	9622 (96.3%)	
Perceived body image	Very thin	1472 (7.9%)	911 (9.1%)	59.003 (<0.001 ***)
	Slightly thin	3970 (21.4%)	2062 (20.6%)	
	Normal	8080 (43.6%)	4139 (41.4%)	
	Slightly fat	4106 (22.3%)	2210 (22.2%)	
	Very fat	898 (4.8%)	666 (6.7%)	
Body mass index (kg/m^2^)	Underweight	10,395 (56.1%)	5636 (56.4%)	1.376 (0.711)
	Normal	5525 (29.8%)	2918 (29.2%)	
	Overweight	1221 (6.6%)	666 (6.7%)	
	Obese	1385 (7.5%)	768 (7.7%)	
Experience of being bullied	Yes	592 (3.2%)	372 (3.7%)	5.559 (0.019 *)
	No	17,934 (96.8%)	9616 (96.3%)	
Need for counseling for school life problems	Yes	358 (1.9%)	204 (2.0%)	0.407 (0.533)
	No	18,168 (98.1%)	9784 (98.0%)	
Request for counseling for distress	Yes	204 (1.1%)	144 (1.4%)	6.244 (0.014 *)
	No	18,322 (98.9%)	9844 (98.6%)	

Note: * *p* < 0.05, *** *p* < 0.001; assessed using chi-squared tests.

**Table 3:** Association between participation in physical activity and lifestyle habits.

Variable		Odds Ratio	95% Confidence Interval	*p*
Breakfast intake	Always	1.160	1.057–1.272	0.002 **
	Usually	1.062	0.963–1.171	0.231
	Usually not	0.955	0.853–1.070	0.426
	Never	Reference		
Amount of sleep	Less than 6 h	0.836	0.709–0.986	0.034 *
	6–7 h	0.692	0.632–0.758	<0.001 ***
	7–8 h	0.767	0.726–0.810	<0.001 ***
	More than 8 h	Reference		
TV viewing	Yes	0.831	0.786–0.878	<0.001 ***
	No	1.000		
Gaming/Internet use	Yes	0.770	0.729–0.813	<0.001 ***
	No	Reference		
Thoughts about running away from home	Yes	1.099	0.958–1.260	0.178
	No	Reference		
Perceived body image	Very thin	1.138	0.978–1.323	0.094
	Slightly thin	1.429	1.247–1.637	<0.001 ***
	Normal	1.487	1.312–1.686	<0.001 ***
	Slightly fat	1.400	1.242–1.579	<0.001 ***
	Very fat	Reference		
Body mass index (kg/m^2^)	Underweight	0.945	0.842–1.061	0.341
	Normal	0.980	0.878–1.093	0.712
	Overweight	0.962	0.842–1.100	0.574
	Obesity	Reference		

Notes: * *p* < 0.05, ** *p* < 0.01, *** *p* < 0.001; assessed using multivariate logistic regression analysis adjusted for sex.

**Table 4:** Association between participation in physical activity and school life factors.

Variable		Odds Ratio	95% Confidence Interval	*p*
Experience of being bullied	Yes	0.878	0.764–1.008	0.065
	No	Reference		
Need for counseling for school life problems	Yes	1.125	0.934–1.355	0.215
	No	Reference		
Need for counseling for distress	Yes	0.802	0.635–1.013	0.064
	No	Reference		

Note: assessed using multivariate logistic regression analysis adjusted for sex.

## Data Availability

Publicly available datasets were analyzed in this study. These data can be found here: “https://schoolhealth.kr/web/srs/selectPrivacyAgree.do?popup=true#none (accessed on 7 February 2025)”.
